# Altered Mechanical Properties of Astrocytes Lacking MLC1: Implications for the Leukodystrophy MLC

**DOI:** 10.1002/glia.70104

**Published:** 2025-12-02

**Authors:** Quinty Bisseling, Emma M. J. Passchier, Freya M. Kirwan, Nelda Antonovaite, Serena Camerini, Maria S. Brignone, Amélie Freal, Huibert D. Mansvelder, Elena Ambrosini, Marjo S. van der Knaap, Rogier Min

**Affiliations:** ^1^ Department of Child Neurology Amsterdam Leukodystrophy Center, Emma Children's Hospital, Amsterdam University Medical Center, Amsterdam Neuroscience Amsterdam the Netherlands; ^2^ Department of Integrative Neurophysiology Center for Neurogenomics and Cognitive Research, Vrije Universiteit Amsterdam, Amsterdam Neuroscience Amsterdam the Netherlands; ^3^ Optics11 Life Amsterdam the Netherlands; ^4^ Core Facilities (FAST), Istituto Superiore di Sanità Rome Italy; ^5^ Department of Neuroscience Istituto Superiore di Sanità Rome Italy; ^6^ Department of Functional Genomics, Center for Neurogenomics and Cognitive Research Amsterdam University Medical Center, Amsterdam Neuroscience Amsterdam the Netherlands; ^7^ Department of Neurology, Epileptology Uniklinik RWTH Aachen Aachen Germany

**Keywords:** astrocyte, cytoskeleton, focal adhesions, leukodystrophy, mechanobiology

## Abstract

Loss of function of the astrocyte protein MLC1 causes Megalencephalic Leukoencephalopathy with subcortical Cysts (MLC), a leukodystrophy characterized by white matter edema and slow neurological deterioration. MLC1 dysfunction leads to swelling of perivascular astrocyte endfeet and an impaired attachment of endfeet to blood vessels. In isolated primary astrocytes, loss of MLC1 hinders recovery of astrocytes from cell swelling, but the cellular function of MLC1 is not completely understood. MLC1 modulates gating of mechanosensitive ion channels involved in volume regulation. The cytoskeleton plays a crucial role in cell volume regulation, and interactions between the cytoskeleton and cell membrane affect the properties of mechanosensitive ion channels. Therefore, we investigated whether primary *Mlc1*‐null mouse astrocytes show a disruption in their mechanical properties. We measured the mechanical properties of cultured primary astrocytes with an indentation technique and demonstrated that *Mlc1*‐null astrocytes are softer than wild‐type astrocytes. Proteomic analysis and western blots confirmed dysregulation of several cytoskeleton‐related pathways in *Mlc1*‐null astrocytes. Confocal imaging revealed that the organization of the actin cytoskeleton and microtubule acetylation are unaffected. Instead, in *Mlc1*‐null astrocytes we observed a decrease in the number of focal adhesions, which aid in relaying mechanical forces between the cytoskeleton, cell membrane, and the extracellular matrix (ECM). Inversely, overexpression of MLC1 in HeLa cells led to an increase in focal adhesions. Together, our findings reveal that the mechanical properties of *Mlc1*‐null astrocytes are altered, and that disrupted cytoskeleton‐membrane‐ECM interactions potentially play a role in the disease. Modulators of astrocyte mechanobiology might therefore hold promise for MLC therapy development.

## Graphical abstract

- Mechanical properties of *Mlc1*‐null astrocytes are altered.
- Cytoskeleton‐related pathways are dysregulated in *Mlc1*‐null astrocytes.
- *Mlc1*‐null astrocytes show alterations in focal adhesions, which link the cytoskeleton to the extracellular matrix.

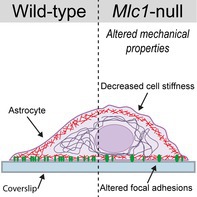

## 1 Introduction

Astrocytes play a crucial role in the regulation of fluid flow in the brain. They form specialized compartments called endfeet, which surround the entire brain microvasculature (Mathiisen et al. 2010). Endfeet are equipped with channels, pumps and membrane proteins essential for brain ion and water homeostasis. Dysfunction of endfeet leads to neurological diseases (Min and van der Knaap 2018), such as the leukodystrophy Megalencephalic Leukoencephalopathy with subcortical Cysts (MLC, OMIM 604004).

MLC is characterized by disturbed brain ion and water homeostasis (Ridder et al. 2011; van der Knaap et al. 2012). Patients have macrocephaly due to chronic brain white matter edema, and they have epilepsy, mild cognitive disability and progressive motor impairment (van der Knaap et al. 1995; Singhal et al. 1996). In MLC, astrocyte endfeet are swollen and fluid‐filled vacuoles can be found in myelin and, to a lesser degree, in astrocyte endfeet (van der Knaap et al. 2012; Dubey et al. 2015). In most MLC patients, the disease is caused by a loss of function of the membrane protein MLC1 (Leegwater et al. 2001), which is highly expressed in astrocyte endfeet (Boor et al. 2005). Defects in several other endfoot proteins that interact with MLC1 also cause MLC, specifically the glial cell adhesion molecule GlialCAM, the orphan G protein‐coupled receptor GPRC5B and the main water channel of the brain, Aquaporin‐4 (AQP4) (López‐Hernández, Ridder, et al. 2011; Passchier et al. 2023).

All four MLC‐related proteins have been linked to astrocyte volume regulation. Primary *Mlc1*‐null mouse astrocytes, as well as patient lymphoblasts, show a defect in regulatory volume decrease (RVD), the process that allows cells to recover from osmotic swelling. This defect has been attributed to dysfunction of mechanosensitive ion channels such as the transient receptor potential cation channel subfamily V member 4 (TRPV4) and the volume‐regulated anion channel (VRAC) (Ridder et al. 2011; Lanciotti et al. 2012; Dubey et al. 2015; Jentsch 2016; Passchier et al. 2023). The exact function of MLC1 and the mechanisms by which MLC1 modulates ion channel activity and astrocyte volume regulation remain unclear (Elorza‐Vidal et al. 2018).

Volume regulation relies on cellular mechanical properties, which are determined by the cytoskeleton and its interactions with the membrane and the extracellular matrix (ECM). The cytoskeleton consists of intermediate filaments, microtubules and actin. Actin filaments can interact with macromolecular complexes, like focal adhesions (FAs), providing a direct link between the intracellular cytoskeleton and the ECM. The cytoskeleton plays an important role in astrocyte volume regulation through modulation of mechanosensitive volume‐regulated ion channels (Levitan et al. 1995; Lascola and Kraig 1996; Lascola et al. 1998; Turovsky et al. 2020). Several studies suggest a role for MLC‐related proteins in modulating cytoskeleton‐membrane‐ECM interactions, thereby affecting cellular mechanical properties. MLC1 and GlialCAM are predominantly present at astrocyte‐astrocyte junctions, where cis and trans interactions between GlialCAM proteins support cell‐to‐cell adhesion (Teijido et al. 2007; López‐Hernández, Ridder, et al. 2011; López‐Hernández, Sirisi, et al. 2011). MLC1 colocalizes with the actin cytoskeleton (Duarri et al. 2011) and is associated with the dystrophin‐associated glycoprotein complex (DAGC), which supports adhesion of perivascular endfeet to the ECM (Boor et al. 2007; Ambrosini et al. 2008). *Mlc1*‐null mouse tissue shows a decrease in perivascular endfeet coverage, with endfeet not being adequately attached to the vasculature (Gilbert et al. 2021). Both MLC1 and AQP4 interact with TRPV4, which is directly connected to the actin cytoskeleton (Benfenati et al. 2011; Lanciotti et al. 2012; Jo et al. 2015). MLC1 negatively regulates actin branching by binding the Arp2/3 complex, which regulates actin nucleation (Hwang et al. 2019). Besides directly interacting with MLC1, the Arp2/3 complex transiently binds vinculin, which promotes FA formation (DeMali et al. 2002; Chorev et al. 2014). Vinculin is a large scaffolding protein that resides in the force‐transduction layer of FAs and links the ECM to the actin cytoskeleton (Kanchanawong et al. 2010). Thus, multiple studies indicate interactions between MLC1 and different components that are important in regulating the mechanical properties of cells.

How the loss of MLC1 function affects astrocyte mechanobiology has not been studied. The aim of this study is to investigate the mechanical properties of cultured primary astrocytes isolated from an MLC mouse model. We hypothesize that the loss of function of MLC1 alters the structural integrity of astrocytes by disturbing cytoskeleton‐membrane‐ECM interactions.

## 2 Materials and Methods

### 2.1 Animals

Primary astrocyte cell cultures were obtained from wild‐type and transgenic *Mlc1*‐null mice. All mice had a C57Bl/6J background. Generation of *Mlc1*‐null mice is described in (Dubey et al. 2015). Wild‐type and *Mlc1*‐null mice were obtained by homozygous breeding of *Mlc1* ^wt/wt^ × *Mlc1* ^wt/wt^ or *Mlc1* ^null/null^ × *Mlc1* ^null/null^ mice, respectively. Breeding pairs were regularly refreshed from heterozygous breeding to limit drift in genetic background. Experimental procedures involving mice were in strict compliance with animal welfare policies of the Dutch government and were approved by the Institutional Animal Care and Use Committee of the Amsterdam University Medical Center, location AMC, Amsterdam, or of the Vrije Universiteit Amsterdam, depending on the location of experiments.

### 2.2 Isolation of Primary Astrocytes From Mice

Cortical primary astrocytes were isolated from neonatal wild‐type and *Mlc1*‐null mice on postnatal day 6 to day 9 (p6–p9) (protocol adapted from (McCarthy and de Vellis 1980)). Brains were isolated from the skull and cerebral cortices were dissected by removing the olfactory bulb, cerebellum, midbrain and meninges. This was done in ice‐cold, sterile Hanks' Balanced Salt Solution without calcium and magnesium, with Phenol Red (HBSS^−/−^; Gibco, 14170‐088) supplemented with 1% Penicillin Streptomycin (Pen Strep; Gibco, 15140‐122). The obtained neocortical tissue was minced with a sterile scalpel and incubated in TrypLE Express (1X; Gibco, 12605‐010) supplemented with DNase I (40 μg/mL; Roche, 11284932001) in a rotator device for 25 min at 37°C. The cell suspension was centrifuged (475 × *g*, 3 min) at room temperature (RT) and the supernatant was removed. The pellet was resuspended in complete primary astrocyte medium (DMEM/F‐12 (1:1) with GlutaMAX‐I and Phenol Red (Gibco, 31331‐028), 10% Fetal Bovine Serum (FBS; Gibco, 10270‐106), 1% Sodium Pyruvate (100 mM; Gibco, 11360‐039) and 1% Pen Strep) and centrifuged again (475 × *g*, 3 min, RT). For mechanical dissociation, the cell pellet was titrated in complete primary astrocyte medium using a 5 and 2 mL pipette, respectively. In between and after the titration steps, the cell suspension was centrifuged (475 × *g*, 3 min, RT) and resuspended in complete primary astrocyte medium. The cell suspension was filtered through a 70 μm Nylon Cell Strainer (Corning, 431751) and centrifuged (475 × *g*, 5 min, RT). Finally, the cell pellet was resuspended in complete primary astrocyte medium and transferred into poly‐L‐lysine (PLL)‐coated (0.1 mg/mL for 1–2 h at 37°C; Sigma, P2636) T75 flasks and incubated in a humidified CO_2_ incubator (37°C/5% CO_2_). Until > 80% confluency was reached (~1 week), cells were washed with Dulbecco's Phosphate Buffered Saline (DPBS; Gibco, 14190‐094) and medium was changed every 2–3 days. At > 80% confluency, contaminating oligodendrocytes, microglia and precursor cells were dislodged overnight (ON) by orbital shaking (180 RPM; VWR, 89032‐088). The next day, flasks were vigorously shaken by hand for 30 s and washed in DPBS three times. Complete primary astrocyte medium was added, and cells were used in experiments or maintained for a week maximum in a humidified CO_2_ incubator (37°C/5% CO_2_) with medium changes every 3 days.

### 2.3 Cell Culture and Treatments

Primary astrocytes were washed with DPBS and incubated in TrypLE Express for 5 min at 37°C. The flask was tapped furiously by hand, and the cells were resuspended in complete primary astrocyte medium and used for plating or preparation of cell pellets.

*For indentation experiments*, primary astrocytes were seeded in uncoated 24‐well plates (Biofil, TCP‐011‐024) at a density of 15,000 cells per well and used for experiments between 24 and 48 h after plating. Before the start of the experiment, cells were treated with cytochalasin D (2, 5 or 10 μM; Sigma‐Aldrich, C2618, 5 mg/mL in DMSO) diluted in complete primary astrocyte medium for 1 h at 37°C. After treatment medium was replaced with 1 mL isotonic solution in which experiments were performed. The isotonic solution contained (in mM): 140 NaCl, 4 KCl, 2 MgCl_2_, 2 CaCl_2_, 10 HEPES, 5 D(+)‐glucose (pH adjusted to 7.4 with NaOH and osmolality adjusted to 300 mOsm/kg).

*For the cell pellet preparation for mass spectrometry and western blots*, primary astrocytes were collected into a 50 mL tube after resuspension in complete primary astrocyte medium. DPBS was added to the cell suspension to fill up the tube to 50 mL. The cell suspension was washed with DPBS 3 times by spinning down the cells to form a cell pellet (400 × *g*, 5 min, RT), then removing the supernatant and adding DPBS after each round. The cell pellets were resuspended in 10 mL DPBS, and cells were counted. After spinning down again, the cell pellet was resuspended and transferred to freezer vials (VWR, 479‐1256) at 2 × 10^6^ cells/mL and spun down (400 × *g*, 5 min, RT). Excess DPBS was removed, and the cell pellets were frozen at −20°C and after 24 h stored at −80°C. Cell pellets were shipped on dry ice. Each cell pellet represents one mouse brain.

*For immunofluorescence staining*, primary astrocytes were seeded on PLL‐coated (0.1 mg/mL for 1–2 h at 37°C) glass coverslips (13 mm, VWR, 631‐1578) in a 24‐well plate at 15,000 cells per well. A total of 48 h after plating the cells were washed three times with DPBS, fixed in DPBS with 2% paraformaldehyde (PFA; Electron Microscopy Sciences, 15710‐S) for 15 min at RT. For staining of microtubules, cells were fixed with ice‐cold methanol for 5 min at −20°C. After removal of methanol and washing with DPBS three times, cells were additionally fixed with DPBS with 2% PFA for 5 min at RT. Coverslips were stored at 4°C in sodium azide 0.05% solution (Merck, RTC000068) to prevent bacterial growth, and used within 4 weeks.

HeLa‐S3 cells were cultured in Dulbecco's Modified Eagle Medium (DMEM; Gibco, 41966‐029), high glucose, pyruvate, supplemented with 10% FBS and 1% Pen Strep in a humidified CO_2_ incubator (37°C/5% CO_2_), and passaged 1:10 dilution two times per week. HeLa‐S3 cells were plated on PLL‐coated (0.1 mg/mL for 1–2 h at 37°C) 13 mm glass coverslips in a 24‐well plate at 5000 cells per well. Cells were transfected 24 h after plating with pEGFPN1‐hMLC1, containing a human MLC1 with a C‐terminal EGFP (Boor et al. 2005), using FuGENE HD (Promega, E2311) according to the manufacturer's instructions (DNA:FuGENE ratio 1:3), mock conditions following the same transfection protocol but without adding DNA. Cells were washed with DPBS three times 24 h post‐transfection, fixed in DPBS with 2% PFA for 15 min at RT and stored at 4°C in sodium azide 0.05% solution (Merck, RTC000068), to prevent bacterial growth, and used within 4 weeks.

### 2.4 Indentation Setup and Protocol

**FIGURE 1:**
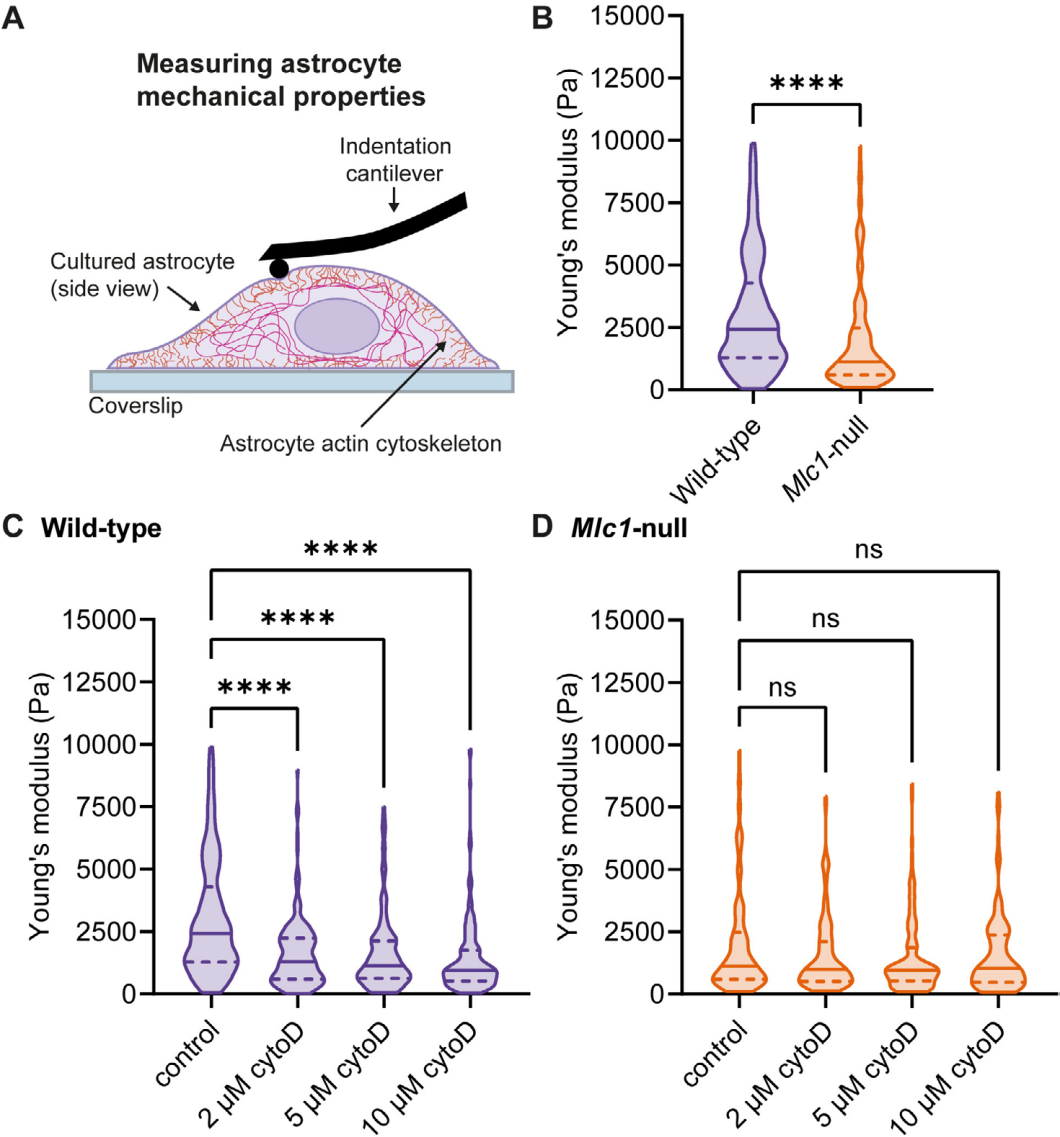
Characterization of mechanical properties of primary wild‐type and *Mlc1*‐null astrocytes reveal that *Mlc1*‐null astrocytes are softer than wild‐type astrocytes. (A) Schematic representation of the experimental indentation setup. (B) Single‐cell indentation measurements of wild‐type astrocytes (purple) and *Mlc1*‐null astrocytes (orange) in Young's modulus, in pascal (Pa) (wild‐type: 2422 (2998) Pa, *n* = 273/*N* = 3, *Mlc1*‐null: 1121 (1181.2) Pa, *n* = 184/*N* = 3, adj. *p* < 0.0001). (C, D) Modulating the cytoskeleton with an actin polymerization inhibitor, cytochalasin D (cytoD; 2, 5 or 10 μM), in wild‐type (C; wild‐type control: 2422 (2998) Pa, *n* = 273/*N* = 3, wild‐type 2 μM: 1293 (1643.2) Pa, *n* = 178/*N* = 3, adj. *p* < 0.0001, wild‐type 5 μM: 1135 (1497) Pa, *n* = 175/*N* = 3, adj. *p* < 0.0001, wild‐type 10 μM: 942 (1241.5) Pa, *n* = 200/*N* = 3, adj. *p* < 0.0001), and in *Mlc1*‐null astrocytes (D; *Mlc1*‐null control: 1121 (1881.2) Pa, *n* = 184/*N* = 3, *Mlc1*‐null 2 μM: 987 (1603) Pa, *n* = 127/*N* = 3, adj. *p* = 0.6872, *Mlc1*‐null 5 μM: 963.5 (1329.5) Pa, *n* = 128/*N* = 3, adj. *p* = 0.1727, *Mlc1*‐null 10 μM: 1037 (1896) Pa, *n* = 129/*N* = 3, adj. *p* = 0.5601). *N* = 3 mice per genotype. All data are presented as median and interquartile range (IQR) values.

Mechanical properties of cultured primary astrocytes were measured with a nanoindenter (Pavone, Optics11 Life, Amsterdam, the Netherlands), using an established protocol (Antonovaite et al. 2020). The 24‐well plate containing primary astrocytes was placed into the nanoindenter, where it was held at 37°C. The nanoindenter uses a cantilever‐based force sensor equipped with a glass sphere that makes contact with the sample (Antonovaite et al. 2023). The sensor is moved down into the well using a piezo, while the lateral position of the sphere is monitored via an integrated inverted microscope. Upon touching the sample with the sphere, bending of the cantilever is measured via an optical fiber and thus applied force and indentation depth are measured in order to extract the Young's modulus. Single‐cell indentation measurements were performed once per cell, at a single position next to the nucleus, using a cantilever stiffness of *k* = 0.025 N/m and a 3 μm radius spherical tip (see schematic illustration in Figure 1A). All data from a plate were obtained within 3 h after the plate was taken from the incubator. Data were analyzed in Matlab by fitting the initial load‐indentation curve (< 1 μm depth) with the Hertz model to extract Young's modulus E (Antonovaite et al. 2020).

### 2.5 Mass Spectrometry

Total protein extracts derived from wild‐type and *Mlc1*‐null astrocyte pellets (prepared as described in Section 2.3) were obtained as previously described (Lanciotti et al. 2012). Briefly, pellets were lysed in a buffer containing 1% Triton X‐100, 0.5% sodium deoxycholate, 150 mM NaCl, 10 mM HEPES (pH 7.4) and a protease (Sigma‐Aldrich, P8340) and phosphatase (Roche Diagnostics, Mannheim, Germany) inhibitor cocktail. Lysates were passed through a 26‐gauge needle 15 times, incubated on ice for 20 min and centrifuged (14,000 × *g*, 20 min, 4°C). Protein loading content was quantified using a bicinchoninic acid (BCA) protein assay kit (Thermo Fisher Scientific, Italy). Samples obtained were analyzed in triplicate: 50 μg of cell lysates derived from each sample was sonicated in an ultrasonic bath for 20 min, incubated in 30 mM Tris(2‐carboxylethyl)phosphine (TCEP) for 10 min and then in 20 mM iodoacetamide for 30 min in the dark at RT. A total of 200 μL of acetone, methanol, and ethanol mixture (25/25/50) was added and the proteins were precipitated ON at −20°C. The protein pellet was centrifuged (14,000 × *g*, 15 min) and digested with 12 ng/μL trypsin (Promega Corporation, WI, United States) in 25 mM ammonium bicarbonate and 1 M urea at 37°C ON. Formic acid was added to a final concentration of 0.1% to stop the enzymatic digestion and an aliquot of the resulting peptide solution was injected in an Ultimate 3000 UHPLC (Dionex, Thermo Fisher Scientific, Waltham, Massachusetts, USA) coupled with an Orbitrap Fusion Tribrid mass spectrometer (Thermo Fisher Scientific, Waltham, Massachusetts, USA). Peptides were desalted on a trap column (Acclaim PepMap 100 C18, Thermo Fisher, Scientific Waltham, Massachusetts, USA) and then separated on a 45‐cm‐long silica capillary (Silica Tips FS 360‐75‐8, New Objective, MA, United States), packed in‐house with a C18, 1.9 μm, 100 Å resin (Michrom BioResources, CA, United States).

The analytical separation ran for 180 min using a gradient of buffer A (5% acetonitrile and 0.1% formic acid) and buffer B (95% acetonitrile and 0.1% formic acid). The gradient started with 5% of buffer B, which rose to 6% in 5 min, to 32% in 130 min, to 55% in 25 min, and up to 80% in 4 min. Then washing and equilibrating steps were added until a total of 180 min run time. Full‐scan MS data were acquired in the 350–1550 m/z mass range in the Orbitrap with 120K resolution. Data‐dependent acquisition was performed in top‐speed mode (3 s long maximum total cycle): the most intense precursors with charge > 1 were selected through a monoisotopic precursor selection (MIPS) filter, quadrupole isolated and fragmented by 30% higher‐energy collision dissociation (HCD). Fragment ions were analyzed in the ion trap with a rapid scan rate. Raw data were analyzed by the software Proteome Discoverer 2.4 (Thermo Fisher Scientific) using the reviewed version of the UniProtKB/Swiss‐Prot mouse (taxonomy 10,090) database containing 17,090 sequences. Spectral matches were filtered using the Percolator node, with 1% false discovery rate (FDR) based on *q* values. Only master proteins were considered and specific trypsin cleavages with two miss‐cleavages were admitted. Cysteine carbamydomethylation was set as a static modification, while methionine oxidation and N‐acetylation on protein terminus were set as variable modifications. 10 ppm and 0.6 Da tolerance were considered for MS and MS/MS data assignment, respectively. Quantification was based on precursor ion intensity of unique and razor peptides and abundance values were normalized on total peptide amount.

Quantitative results were analyzed by Proteome Discoverer: protein ratio calculation was based on pairwise ratio. Proteins were filtered considering only those quantified at least twice in one group. Of this group, proteins were considered up‐ or downregulated in *Mlc1*‐null vs wild‐type astrocytes if the log_2_ ratio *Mlc1*‐null/wild‐type was greater than 0.6 or lower than −0.6, respectively, with an adjusted *p*‐value of < 0.05. Principal component analysis (PCA) was obtained using SRplot (Tang et al. 2023), including only proteins detected in all samples (*n* = 5205). The biological process of enrichment analysis on differentially modulated proteins was conducted by using the WEB‐based GEne SeT AnaLysis Toolkit (Webgestalt) (Liao et al. 2019), looking at the top 10 level categories enriched with 0.05 FDR evaluated by Benjamini correction. Proteins involved in cytoskeleton organization based on Webgestalt analysis were analyzed by STRING (Szklarczyk et al. 2023), and clustered with the Markov Cluster Algorithm (Van Dongen 2008). All the proteomics data have been deposited in the ProteomeXchange Consortium via the MassIVE (Mass Spectrometry Interactive Virtual Environment) with the identifier MSV000097012 (Computer Science and Engineering 2025).

### 2.6 Western Blotting (WB)

Total protein extracts from wild‐type and *Mlc1*‐null astrocyte pellets were obtained as described in Section 2.5. After quantification of protein loading content using a BCA protein assay kit (Thermo Fisher Scientific, Italy), equal amounts of proteins (30 μg) were resolved on SDS–PAGE using gradient (4%–12%) pre‐casted gels (Thermo Fisher Scientific, USA), and transferred onto a nitrocellulose membrane using the Trans‐Blot Turbo Transfer System (BioRad). Nitrocellulose membranes were blotted ON at 4°C using the following antibodies: mouse anti‐GPRC5B (1:500; Santa Cruz Biotechnology, USA), rabbit anti‐MLC1 (1:1500; in‐house generated (Ambrosini et al. 2008)), rabbit anti‐MYL6B (1:1000; ABclonal), mouse anti‐GAPDH (1:1000; Santa Cruz Biotechnology, USA), rabbit anti‐ITGB5 (1:1000; Proteintech), rabbit anti‐actin gamma1 (ACTG1; 1:1000; Proteintech), rabbit anti‐AQP4 (1:500; Proteintech), mouse anti‐Vinculin (1:500; Santa Cruz Biotechnology, USA), rabbit anti‐GlialCAM (1:500; Proteintech), rabbit anti‐RhoA (1:1000; Abcam, Cambridge, UK), rabbit anti‐EGFR (1:1000; Abcam), mouse anti‐GFAP (1:2000; BD Transduction Laboratories) and mouse anti‐β‐actin (1:2000; Santa Cruz Biotechnology, USA). After washings in Tris buffered saline (TBS), membranes were incubated with horseradish peroxidase‐conjugated anti‐mouse or anti‐rabbit antibodies (1:5000; Bio‐Rad Laboratories, USA) for 1 h at RT. Immunoreactive bands were visualized using an enhanced chemiluminescence reagent (Pierce, Thermo Fisher Scientific, USA), according to the manufacturer's instructions, and exposed on a Bio‐Rad ChemiDoc XRS system (Bio‐Rad Laboratories, USA). Densitometric analysis of WB experiments were performed using ImageJ software (NIH, USA) and a Bio‐Rad ChemiDoc XRS system. GAPDH protein was used as a loading control.

### 2.7 Immunocytochemistry and Imaging

Plates with fixed primary astrocytes or HeLa‐S3 cells on coverslips (see Section 2.3 for preparation) were taken to RT. After removing the 0.05% sodium azide in the fume hood, coverslips were washed three times with DPBS, taken out of the fume hood and washed again three times with DPBS. Coverslips were treated with blocking buffer (DPBS +5% Normal Goat Serum (NGS; Gibco, 16210‐064) + 0.1% Bovine Serum Albumin (BSA; Sigma‐Aldrich, A4919) + 0.3% Triton X‐100 (Sigma‐Aldrich, X100)) for 1 h at RT for permeabilization of cells (with Triton X‐100) and to block nonspecific binding (with BSA and NGS). Then coverslips were incubated with primary antibodies and/or a fluorescently labeled F‐actin probe, diluted in blocking buffer (4°C, ON), which promotes antibody solubility. The next day coverslips were washed three times with DPBS and stained with secondary antibody diluted in blocking buffer for 1 h at RT. Coverslips were then washed three times with DPBS, and with the third wash stained for 4′,6‐diamidino‐2‐phenylindole (DAPI; 1:2000; Sigma, D9542, 5 mg/mL) diluted in DPBS. Coverslips were washed once with DPBS to remove excess DAPI and mounted on glass slides using ProLong Glass Antifade Mountant mounting medium (Invitrogen, P36984); then, in the dark, stored first at RT for 24 h and then at 4°C until use. The following antibodies were used for staining of vinculin: anti‐vinculin (1:800; Sigma‐Aldrich, V9131, mouse monoclonal antibody IgG1, 5–10 mg/mL), and, as secondary, Alexa Fluor 488 goat IgG anti‐mouse (1:1000; Invitrogen, A‐11001, 2 mg/mL) for the astrocytes, and Alexa Fluor 647 goat IgG anti‐mouse (1:1000; Invitrogen, A‐21236, 2 mg/mL) for the HeLa cells. For staining of F‐actin, a tetramethylrhodamine isothiocyanate (TRITC)‐conjugated phalloidin probe (1 unit; Invitrogen, R415, 300 units) was used. For the microtubule stainings the following antibodies were used: anti‐Acetylated Tubulin antibody (1:5000; Sigma‐Aldrich, T7451, mouse monoclonal antibody IgG2b, ~1 mg/mL) with, as secondary, Alexa Fluor 488 goat IgG anti‐mouse IgG2b (1:1000; Invitrogen, A‐21141, 2 mg/mL), and anti‐α‐Tubulin (1:2000; Sigma‐Aldrich, T5168, mouse monoclonal antibody IgG1, 3–7 mg/mL) with, as secondary, Alexa Fluor 568 goat IgG anti‐mouse IgG1 (1:1000; Invitrogen, A‐21124, 2 mg/mL). For the astrocytes, multiple z‐stacks with a z step size of 0.225 μm were obtained on an inverted Nikon Eclipse Ti2 confocal microscope (Nikon, Japan) with an oil‐immersion objective (Nikon Plan Fluor 40X/NA 1.3/WD 240 μm) and intermediate magnification of 1.5X. Sequentially, DAPI was excited at 406 nm (emission filter: 425–475), vinculin at 488 nm (emission filter: 500–550) and F‐actin at 561 nm (emission filter: 570–616). For HeLa cells, multiple z‐stacks with a z step size of 0.2 μm were obtained on an inverted Leica Stellaris 5 confocal microscope (Leica Microsystems, Germany) with an oil‐immersion objective (HC PL APO CS2 63X/NA 1.4/WD 140 μm). Sequentially, DAPI was excited at 405 nm (emission filter: 430–600), MLC1 at 498 nm (emission filter: 503–556), F‐actin at 553 nm (emission filter: 558–763) and vinculin at 652 nm (emission filter: 657–812). Z‐stack size and image format were determined with the internal Nyquist calculator of the imaging software of the microscope.

### 2.8 Imaging Analysis

Images were analyzed using Fiji (Schindelin et al. 2012). Summation (SUM) and maximum (MAX) intensity projections were made, and cell regions of interest (ROIs) were drawn based on the F‐actin or α‐tubulin channel with the wand tracing tool. Additional square background ROIs were drawn for each image in a region where no specific staining was observed for any of the channels. ROIs were measured for all channels with the multi‐measure tool to obtain morphological and other parameters mentioned below. Values were background‐corrected by subtracting the fluorescence intensity from a background square for each corresponding image. Primary astrocytes with an area larger than 10,000 μm^2^ (*n* = 3) were excluded due to not being completely within the bounds of the image, which made drawing of ROIs not feasible.

Vinculin was used as a general marker for FAs. FA analysis was done following a modified protocol (Güler et al. 2021). MAX intensity projections were split into different channels, and the channel containing the vinculin staining was adjusted into 8‐bit. The image was processed by subtracting the background with Fast Fourier Transformation (FFT) Bandpass Filter. Large structures were filtered down to 20 pixels and small structures to 1 pixel with a 5% tolerance of direction, with autoscaling and saturation selected. The images were thresholded with the Huang method, in black and white with a dark background, to obtain binary outlines of vinculin positive FAs. Next, ROIs of FAs were generated, counted and analyzed with the ‘Analyze Particles’ command (size 10–Infinity in pixels, circularity 0.00–0.99 and showing outlines), with the cell ROI selected to only include FAs in the cell of interest (see Figure 7B for an example). Thresholding alters fluorescence intensity levels of the image; therefore, the generated ROIs of FAs were applied to the original 16‐bit SUM intensity projection image to determine fluorescence intensity values of all individual FAs. These values were background‐corrected as described previously, and values were averaged per cell.

All fluorescence intensity values were obtained from SUM intensity projections and normalized to averaged values of wild‐type cells imaged on the same days. All data were obtained from cells from 3 different mice (2 for microtubule stainings), per genotype. Based on differences in area, data were grouped into three separate groups: 0–1500, 1500–4000 and 4000+ μm^2^. Each group contains cells from all different mice.

### 2.9 Statistics

Data representation and statistical analysis were performed using GraphPad Prism version 10.2.0 for Windows (GraphPad Software, Boston, Massachusetts USA, www.graphpad.com). Data were tested for normality with a Kolmogorov–Smirnov test. Statistical analysis of indentation data was done with a Mann–Whitney *U* test (Figure 1B), and a Kruskal‐Wallis test with Dunn's multiple comparisons test, by comparing all groups with the control group (Figure 1C,D). Statistical analysis of the western blots and imaging data was done with a t‐test or Mann–Whitney *U* test, with a Holm‐Šidák multiple comparisons test for grouped data by comparing wild‐type to *Mlc1*‐null astrocytes in every size group (0–1500, 1500–4000 and 4000+ μm^2^; Figures 4, 5 and 7). Cumulative distributions of the area of wild‐type and *Mlc1*‐null astrocytes were compared with a Kolmogorov–Smirnov test (Figure 4B). Indentation data were reported as median (interquartile range [IQR]); all other data are expressed as mean ± SEM. Statistically significant differences were defined as *p* ≤ 0.05.

## 3 Results

### 3.1 *Mlc1*‐Null Astrocytes Have Altered Mechanical Properties

We investigated the mechanical properties of cultured primary *Mlc1*‐null and wild‐type astrocytes with indentation measurements. To minimize the influence of the plastic substrate on indentation measurements, the chosen measurement point for each cell was close to the nucleus, at its thickest point (Figure 1A) (Antonovaite et al. 2020). To determine cell stiffness, the Young's modulus, or elastic modulus, was determined for individual cells. This measure reflects the resistance of a substance to elastic deformation. *Mlc1*‐null astrocytes had a significantly lower Young's modulus than wild‐type astrocytes (Figure 1B), indicating that loss of MLC1 leads to softer astrocytes. To investigate how disruption of the astrocyte actin cytoskeleton would influence cell stiffness, wild‐type and *Mlc1*‐null astrocytes were treated with an increasing dose of the actin polymerization inhibitor cytochalasin D. Disrupting the actin cytoskeleton caused a significant decrease in the Young's modulus of wild‐type astrocytes for all three used concentrations of cytochalasin D (Figure 1C). Thus, actin cytoskeleton disruption led to softening of wild‐type astrocytes. Treatment of *Mlc1*‐null astrocytes with cytochalasin D did not induce a further decrease in the Young's modulus (Figure 1D). This suggests that the already impaired structural integrity of *Mlc1*‐null astrocytes could not be exacerbated by disruption of the actin cytoskeleton. In conclusion, indentation measurements revealed that *Mlc1*‐null astrocytes are softer than wild‐type astrocytes, and that disruption of the actin cytoskeleton in *Mlc1*‐null astrocytes does not lead to additional softening.

### 3.2 Proteomic Analysis Reveals Alterations of Cytoskeleton‐Related Pathways in *Mlc1*‐Null Astrocytes

**FIGURE 2:**
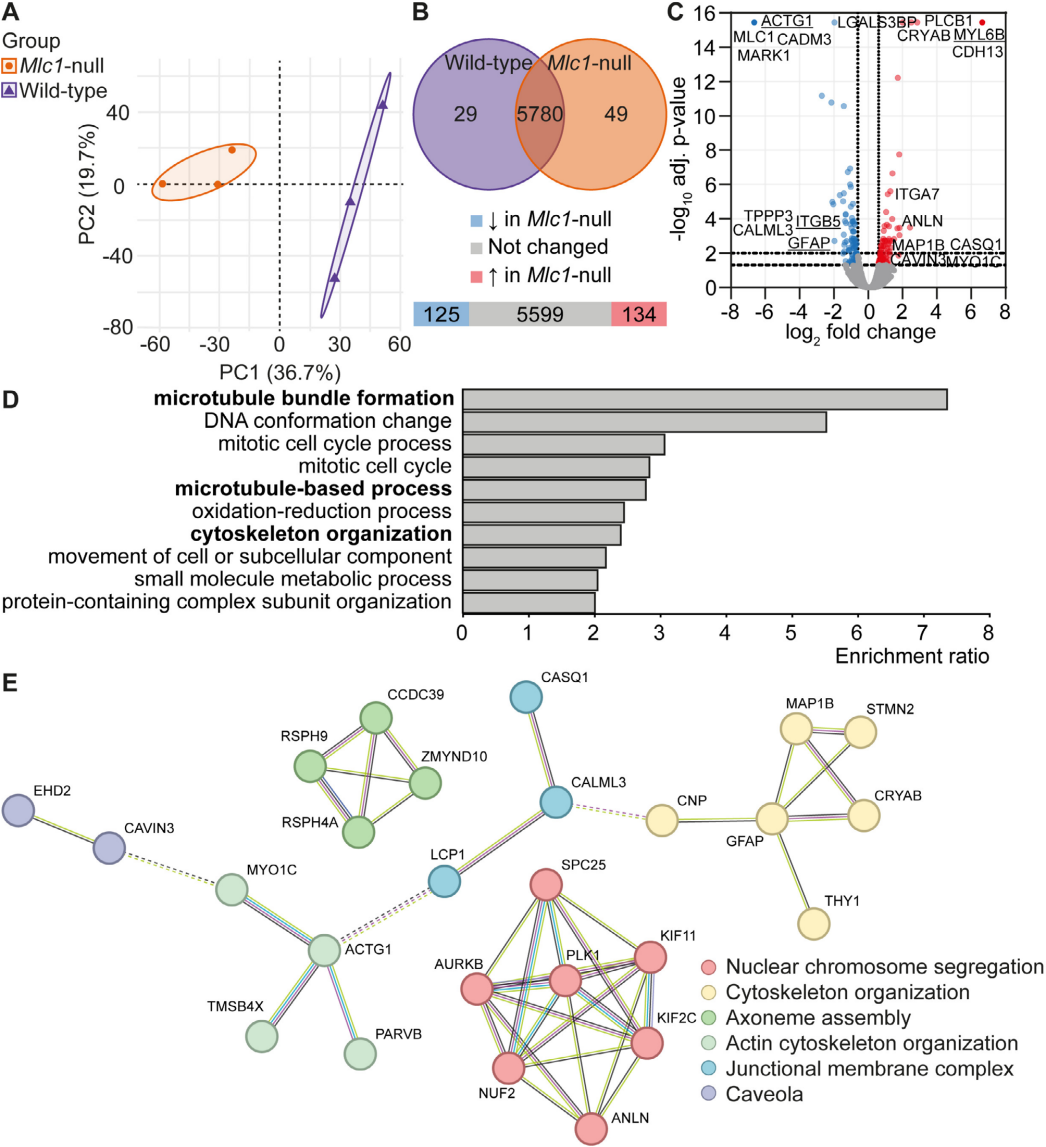
Proteomic analysis shows enrichment of cytoskeleton‐related pathways in *Mlc1*‐null astrocytes. (A) Principal component analysis (PCA) on proteins quantified in all samples (*n* = 5205) shows a clear separation between primary wild‐type (purple) and *Mlc1*‐null (orange) astrocytes. The first component (PC1) separates wild‐type from *Mlc1*‐null astrocytes and explains 36.7% of the variability. The second component (PC2) is representing 19.7% of the variability. (B, C) Venn diagram shows total number of 5858 proteins quantified at least twice in one group. Of these 5858 proteins, 5780 were detected in both wild‐type (purple) and *Mlc1*‐null (orange) astrocytes, 29 only in wild‐type and 49 only in *Mlc1*‐null astrocytes. Differentially expressed proteins in *Mlc1*‐null astrocytes compared to wild‐type astrocytes, based on a fold change > 0.06 or < −0.06 with an adj. *p* < 0.05, are shown in the bar plot (B) and the volcano plot (C). The bar plot shows the number of proteins not changed (*n =* 5599; gray), down‐ (*n* = 125; blue) and upregulated (*n* = 134; red). In the volcano plot, MLC1 and several other proteins related to the cytoskeleton are highlighted. Differential expression of underlined proteins ACTG1, MYL6B, ITGB5 and GFAP were validated using western blotting (see Figures S1 and 3). (D) The WEB‐based GEne SeT AnaLysis Toolkit was used for biological process of enrichment analysis on differentially modulated proteins. The top 10 categories that are enriched with a False Discovery Rate (FDR) of ≤ 0.05 are shown. Three biological processes related to the cytoskeleton are highlighted in bold: Microtubule bundle formation, microtubule‐based process, and cytoskeleton organization. (E) Analysis of protein–protein interaction networks on proteins involved in cytoskeleton organization. *N* = 3 mice per genotype.

We characterized the proteome of cultured primary wild‐type and *Mlc1*‐null astrocytes. PCA showed a clear separation between wild‐type and *Mlc1*‐null samples (Figure 2A). Proteomic analysis led to the identification of 6232 proteins, of which 96% (5960 proteins) could be quantified (for the total list see Supporting Information S1). After data filtering, 5858 proteins were analyzed: 125 proteins were identified as downregulated and 134 as upregulated in *Mlc1*‐null samples compared to wild‐type samples (Supporting Information S2; Figure 2B). These up‐ and downregulated proteins are shown in a volcano plot with several cytoskeleton‐related proteins and other proteins of interest indicated (Figure 2C). Analysis of biological pathways showed significant enrichment for pathways related to the cytoskeleton: Microtubule bundle formation, microtubule‐based process and cytoskeleton organization (Figure 2D; in bold). Proteins from the biological process cytoskeleton organization were further analyzed for protein–protein interaction networks (Figure 2E). As part of the cytoskeleton organization cluster, glial fibrillary acidic protein (GFAP) was downregulated, and the microtubule associated protein 1B (MAP1B) was upregulated in *Mlc1*‐null astrocytes. ACTG1, or γ‐actin, was downregulated and myosin protein MYO1C was upregulated.

**FIGURE 3:**
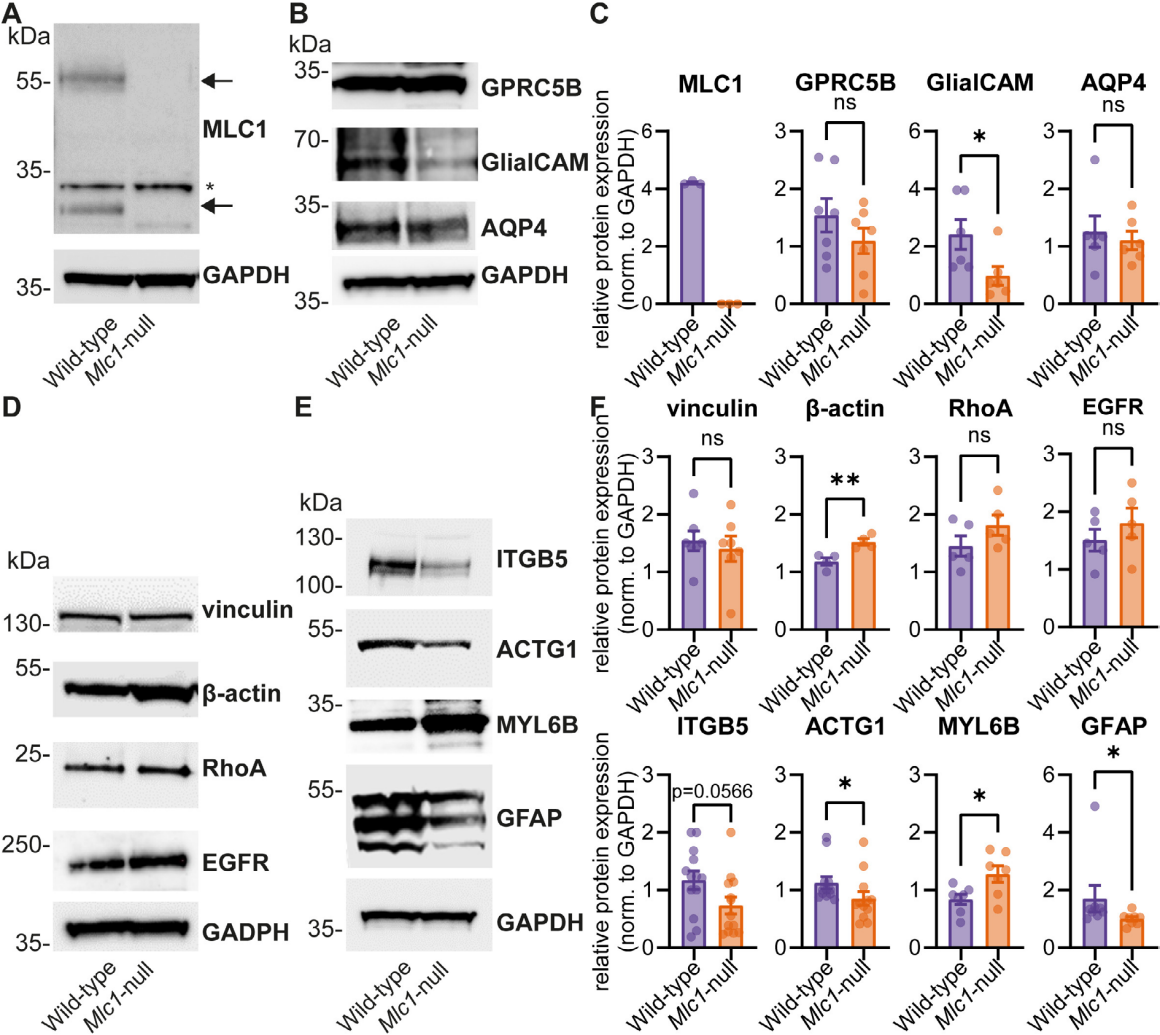
Western blot analysis of protein expression in wild‐type and *Mlc1*‐null astrocytes. (A) Western blot of MLC1 protein in wild‐type and *Mlc1*‐null astrocytes. MLC1 monomeric and dimeric forms are indicated by arrows, and asterisks indicate non‐specific bands recognized by the anti‐MLC1 polyclonal antibody in all samples. (B) Western blots of MLC‐related proteins GPRC5B, GlialCAM and AQP4 in wild‐type and *Mlc1*‐null astrocytes. (C) Quantification of western blots shown in (A) and (B) in wild‐type (purple) and *Mlc1‐*null (orange) astrocytes (relative protein expression; MLC1: Wild‐type: 4.206 ± 0.03968, *Mlc1*‐null: 0 ± 0, *n* = 3, GPRC5B: Wild‐type: 1.541 ± 0.2886, *Mlc1*‐null: 1.095 ± 0.2206, *n* = 7, *p* = 0.2433, GlialCAM: Wild‐type: 2.417 ± 0.5189, *Mlc1*‐null: 0.9753 ± 0.3293, *n* = 6, *p* = 0.041, AQP4: Wild‐type: 1.258 ± 0.2709, *Mlc1*‐null: 1.104 ± 0.1595, *n* = 6, *p* = 0.6667). (D) Western blots of vinculin, β‐actin, RhoA, and EGFR in wild‐type and *Mlc1*‐null astrocytes. (E) Western blots of selected proteins based on proteomics (see Figure S1) in wild‐type and *Mlc1*‐null astrocytes. (F) Quantification of western blots shown in (D) and (E) in wild‐type (purple) and *Mlc1‐*null (orange) astrocytes (relative protein expression; vinculin: Wild‐type: 1.543 ± 0.1725, *Mlc1*‐null: 1.405 ± 0.2204, *n* = 7, *p* = 0.6306, β‐actin: Wild‐type: 1.181 ± 0.06605, *Mlc1*‐null: 1.525 ± 0.05806, *n* = 4, *p* = 0.0079, RhoA: Wild‐type: 1.452 ± 0.1771, *Mlc1*‐null: 1.814 ± 0.1784, *n* = 5, *p* = 0.1869, EGFR: Wild‐type: 1.508 ± 0.1912, *Mlc1*‐null: 1.805 ± 0.2566, *n* = 5, *p* = 0.3802, ITGB5: Wild‐type: 1.171 ± 0.1612, *Mlc1*‐null: 0.7343 ± 0.1468, *n* = 13, *p* = 0.0566, ACTG1: Wild‐type: 1.126 ± 0.1047, *Mlc1*‐null: 0.8544 ± 0.1189, *n* = 12, *p* = 0.0114, MYL6B: Wild‐type: 0.8389 ± 0.08860, *Mlc1*‐null: 1.277 ± 0.1432, *n* = 7, *p* = 0.0232, GFAP: Wild‐type: 1.705 ± 0.4604, *Mlc1*‐null: 1.002 ± 0.07624, *n* = 8, *p* = 0.019). Molecular weight (MW) markers are indicated on the left in kiloDalton (kDa). GAPDH was used as a loading control. Bar graphs show densitometric analysis of protein expression normalized against GAPDH from corresponding samples. All data is presented as mean ± SEM, with dots indicating individual values in bar graphs. Original blots are shown in Figure S2.

A subset of proteins identified in the proteomic analysis was validated by western blotting (Figure S1), including MLC‐related proteins and several cytoskeleton‐related proteins. As expected, MLC1 was absent in *Mlc1*‐null astrocytes, while GPRC5B and AQP4 levels were unchanged (Figure 3A–C). GlialCAM showed a non‐significant downward trend in proteomics but was significantly reduced in western blotting. Consistent with the proteomics, levels of cytoskeleton‐related proteins vinculin, RhoA and EGFR were unchanged, whereas β‐actin showed a small but significant increase not detected in proteomics (Figure 3D–F). For proteins that showed significant differential expression in the proteomic analysis, western blotting confirmed decreased levels of ACTG1 and GFAP and increased levels of MYL6B, myosin light chain 6B. ITGB5, integrin β5, showed a non‐significant downward trend (*p* = 0.0566), consistent with its modest significant fold change in proteomics. Overall, western blotting confirmed the proteomic results for most proteins, supporting the differential regulation of cytoskeleton‐related pathways in *Mlc1*‐null astrocytes.

### 3.3 *Mlc1*‐Null Astrocytes Show No Differences in Morphology, F‐actin Fluorescence Intensity or Microtubule Acetylation

**FIGURE 4:**
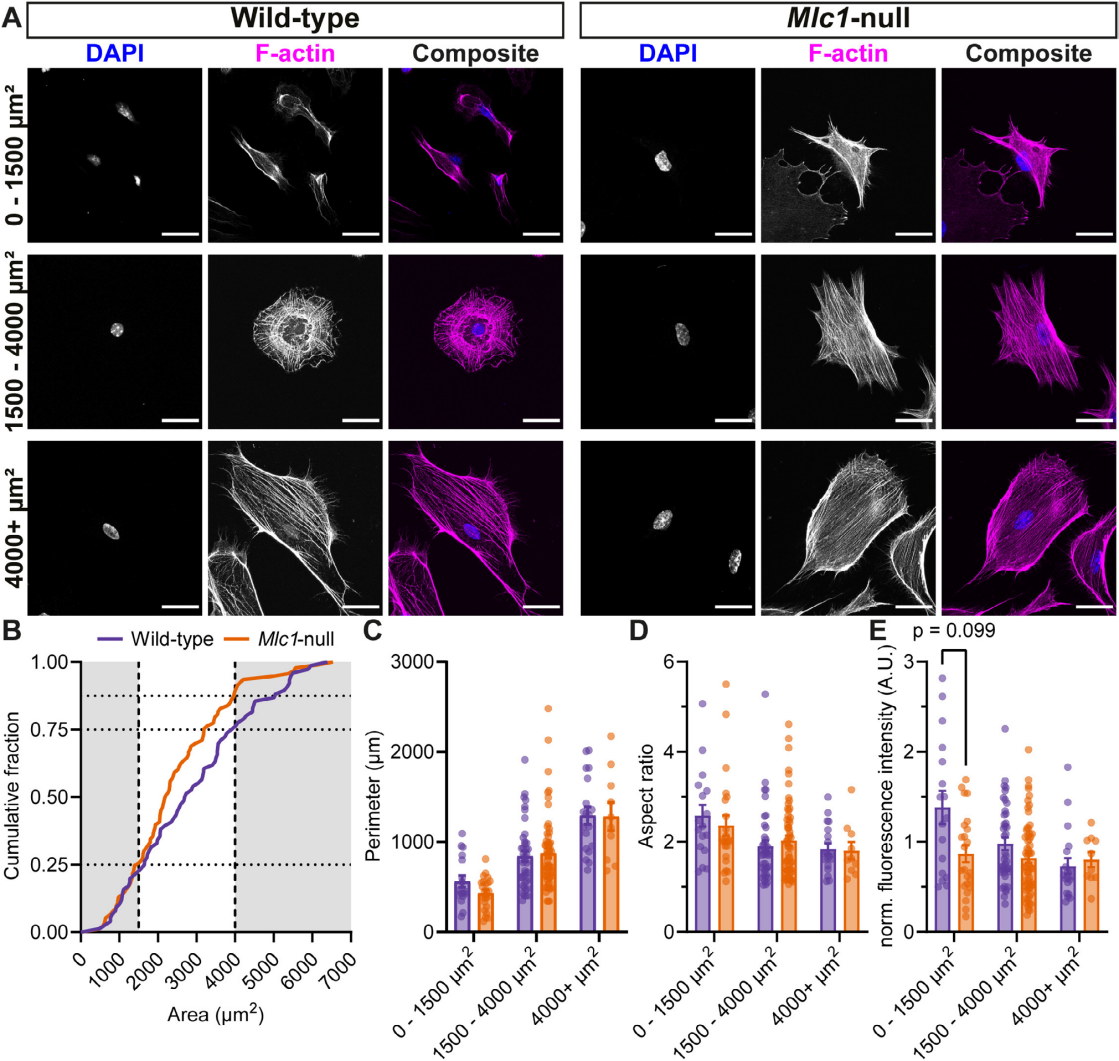
*Mlc1*‐null astrocytes have similar morphological parameters to wild‐type astrocytes, and cells of size 1500–4000 μm^2^ show a trend in decrease of F‐actin fluorescence intensity. (A) Representative confocal images of primary wild‐type and *Mlc1*‐null astrocytes, stained for the nucleus with DAPI (blue) and F‐actin (magenta). The cells are split in three different size groups: 0–1500, 1500–4000 and 4000+ μm^2^. Scale bars: 30 μm. (B) Cumulative distribution function of the area of wild‐type (purple) and *Mlc1*‐null (orange) astrocytes (*D* = 0.1773, approx. *p* = 0.1443). (C, D) Cell morphology parameters perimeter (C) and aspect ratio (D; ratio between height and width of the cells) of wild‐type (purple) and *Mlc1‐*null (orange) astrocytes (perimeter: Wild‐type 0–1500 μm^2^: 560.6 ± 65.6 μm, *Mlc1*‐null 0–1500 μm^2^: 432.5 ± 37.5 μm, adj. *p* = 0.603, wild‐type 1500–4000 μm^2^: 841.1 ± 58.6 μm, *Mlc1*‐null 1500–4000 μm^2^: 873.2 ± 53.3 μm, adj. *p* = 0.902, wild‐type 4000+ μm^2^: 1292.7 ± 96.1 μm, *Mlc1*‐null 4000+ μm^2^: 1281.8 ± 157.1 μm, adj. *p* = 0.902; aspect ratio: Wild‐type 0–1500 μm^2^: 2.58 ± 0.24, *Mlc1*‐null 0–1500 μm^2^: 2.36 ± 0.23, adj. *p* = 0.584, wild‐type 1500–4000 μm^2^: 1.90 ± 0.13, *Mlc1*‐null 1500–4000 μm^2^: 2.03 ± 0.11, adj. *p* = 0.753, wild‐type 4000+ μm^2^: 1.85 ± 0.12, *Mlc1*‐null 4000+ μm^2^: 1.80 ± 0.19, adj. *p* = 0.753). (E) Normalized and background‐corrected fluorescence intensity of F‐actin in wild‐type (purple) and *Mlc1‐*null (orange) astrocytes (wild‐type 0–1500 μm^2^: 1.38 ± 0.18, *Mlc1*‐null 0–1500 μm^2^: 0.87 ± 0.09, adj. *p* = 0.099, wild‐type 1500–4000 μm^2^: 0.98 ± 0.07, *Mlc1*‐null 1500–4000 μm^2^: 0.82 ± 0.05, adj. *p* = 0.15, wild‐type 4000+ μm^2^: 0.73 ± 0.09, *Mlc1*‐null 4000+ μm^2^: 0.80 ± 0.08, adj. *p* = 0.266). Each group contains cells from three different mice per genotype (wild‐type 0–1500 μm^2^: *n* = 17/*N* = 3, *Mlc1*‐null 0–1500 μm^2^: *n* = 23/*N* = 3, wild‐type 1500–4000 μm^2^: *n* = 40/*N* = 3, *Mlc1*‐null 1500–4000 μm^2^: *n* = 60/*N* = 3, wild‐type 4000+ μm^2^: *n* = 19/*N* = 3, *Mlc1*‐null 4000+ μm^2^: *n* = 10/*N* = 3). All data is presented as mean ± SEM, with dots indicating individual values of cells in bar graphs.

**FIGURE 5:**
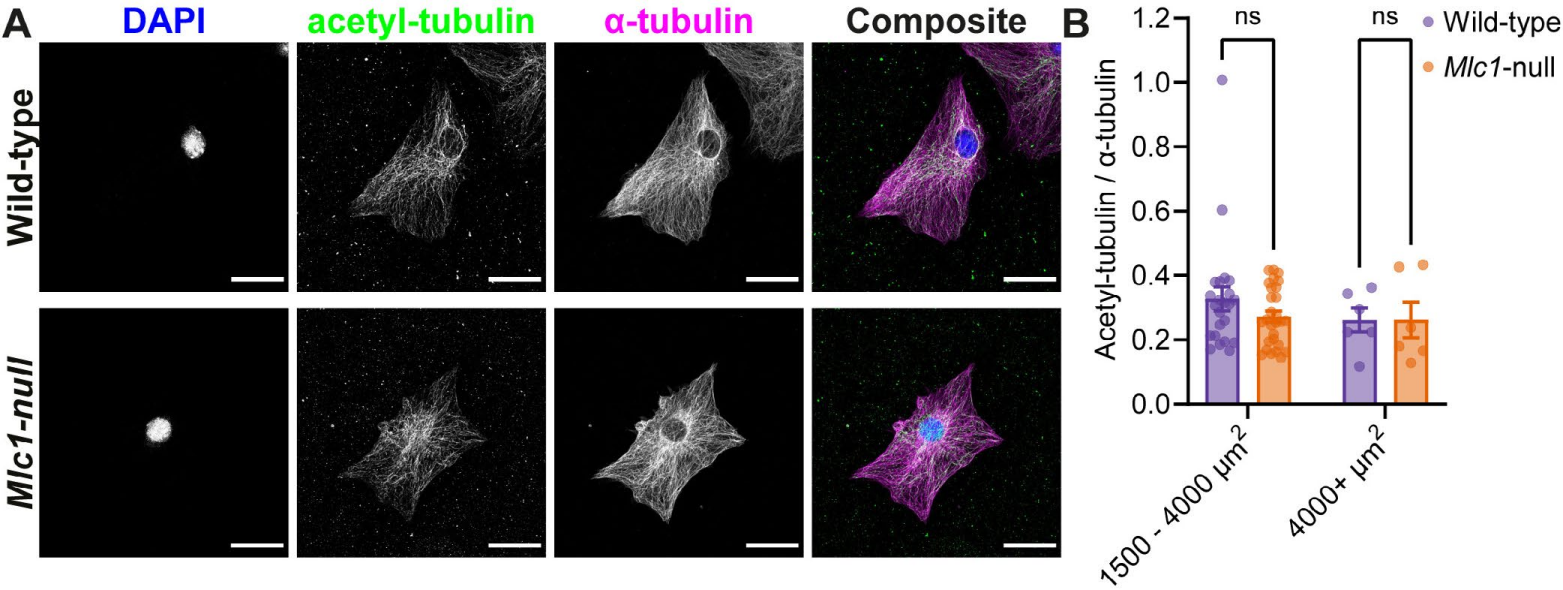
No difference in microtubule acetylation between wild‐type and *Mlc1*‐null astrocytes. (A) Representative confocal images of primary wild‐type and *Mlc1*‐null astrocytes, stained for the nucleus with DAPI (blue), acetylated tubulin (green) and α‐tubulin (magenta). Scale bars: 30 μm. (B) Ratio of background‐corrected fluorescence intensity of acetylated tubulin over α‐tubulin in wild‐type (purple) and *Mlc1‐*null (orange) astrocytes in size groups 1500–4000 and 4000+ μm^2^ (wild‐type 1500–4000 μm^2^: 0.328 ± 0.037, *n* = 23 cells, *Mlc1*‐null 1500–4000 μm^2^: 0.272 ± 0.017, *n* = 28 cells, adj. *p* = 0.530, wild‐type 4000+ μm^2^: 0.262 ± 0.037, *n* = 6 cells, *Mlc1*‐null 4000+ μm^2^: 0.262 ± 0.055, *n* = 6 cells, adj. *p* > 0.999). Size group 0–1500 μm^2^ is not included as there were not enough cells for meaningful analysis. All data is presented as mean ± SEM, with dots indicating individual values of cells in bar graphs.

To investigate the organization of the actin cytoskeleton in cultured primary wild‐type and *Mlc1*‐null astrocytes, we performed immunofluorescence staining of F‐actin. This allowed us to also visualize general cell morphology (Figure 4A). Primary astrocytes were heterogeneous in shape and morphology. On average, wild‐type and *Mlc1*‐null astrocytes span the same area. A cumulative distribution analysis of cell area of wild‐type and *Mlc1*‐null astrocytes showed that about half of the cells were sized between 1500 and 4000 μm^2^ (Figure 4B). We did observe a non‐significant trend towards an overrepresentation of smaller cells in astrocytes isolated from *Mlc1*‐null mice. Since the organization of the cytoskeleton strongly depends on total cell size, we split the cells into three distinct size categories for further analysis. The perimeter and the aspect ratio (ratio of width and height) were similar between wild‐type and *Mlc1*‐null astrocytes in all three categories, indicating comparable morphology (Figure 4C,D). Fluorescence intensity of F‐actin showed no significant differences between wild‐type and *Mlc1*‐null astrocytes. The group of small (0–1500 μm^2^) *Mlc*1‐null astrocytes showed a trend towards lower F‐actin fluorescence levels (Figure 4E). We did not observe any obvious alterations in the distribution of F‐actin throughout the astrocytes. We further investigated changes in the microtubule cytoskeleton using immunofluorescence. Stainings for α‐tubulin and acetylated tubulin (Figure 5A), representing the total microtubules and the long‐lived, stable microtubules pool, respectively (Piperno et al. 1987), revealed no differences between wild‐type and *Mlc1*‐null astrocytes (Figure 5B). Overall, immunofluorescence analysis suggested no strong alterations in the levels or organization of F‐actin nor in the stability of microtubules between wild‐type and *Mlc1*‐null astrocytes.

### 3.4 Alterations in Focal Adhesions in *Mlc1*‐Null Astrocytes and MLC1‐Transfected HeLa Cells

**FIGURE 6:**
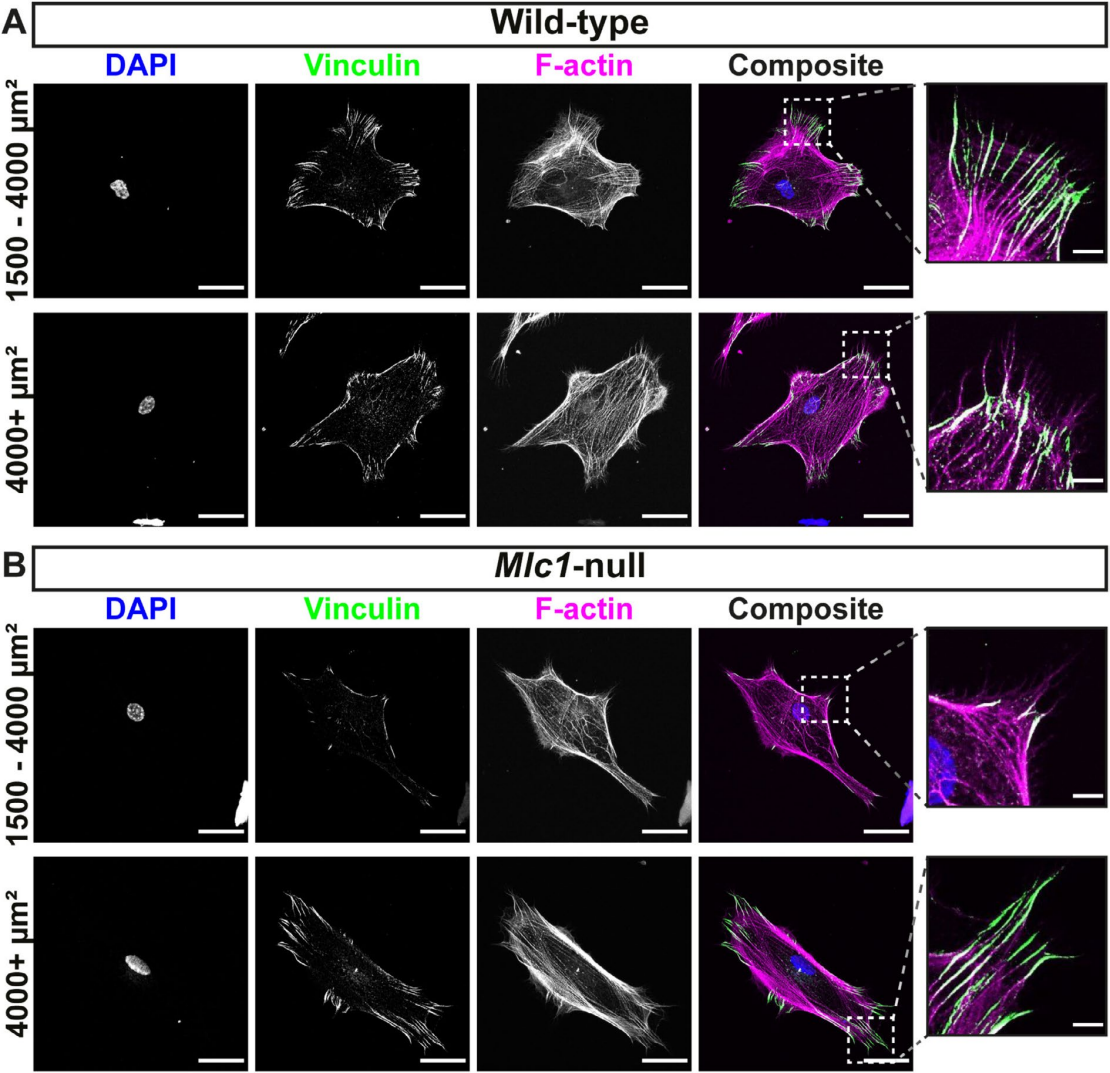
Immunofluorescence staining of vinculin, a focal adhesion protein, in wild‐type and *Mlc1*‐null astrocytes. Representative confocal images of primary wild‐type (A) and *Mlc1*‐null (B) astrocytes in size groups 1500–4000 and 4000+ μm^2^, stained for the nucleus with DAPI (blue), vinculin (green) and F‐actin (magenta). Focal adhesions are shown in the enlarged images. Scale bars: 30 μm and 5 μm for the enlarged images.

**FIGURE 7:**
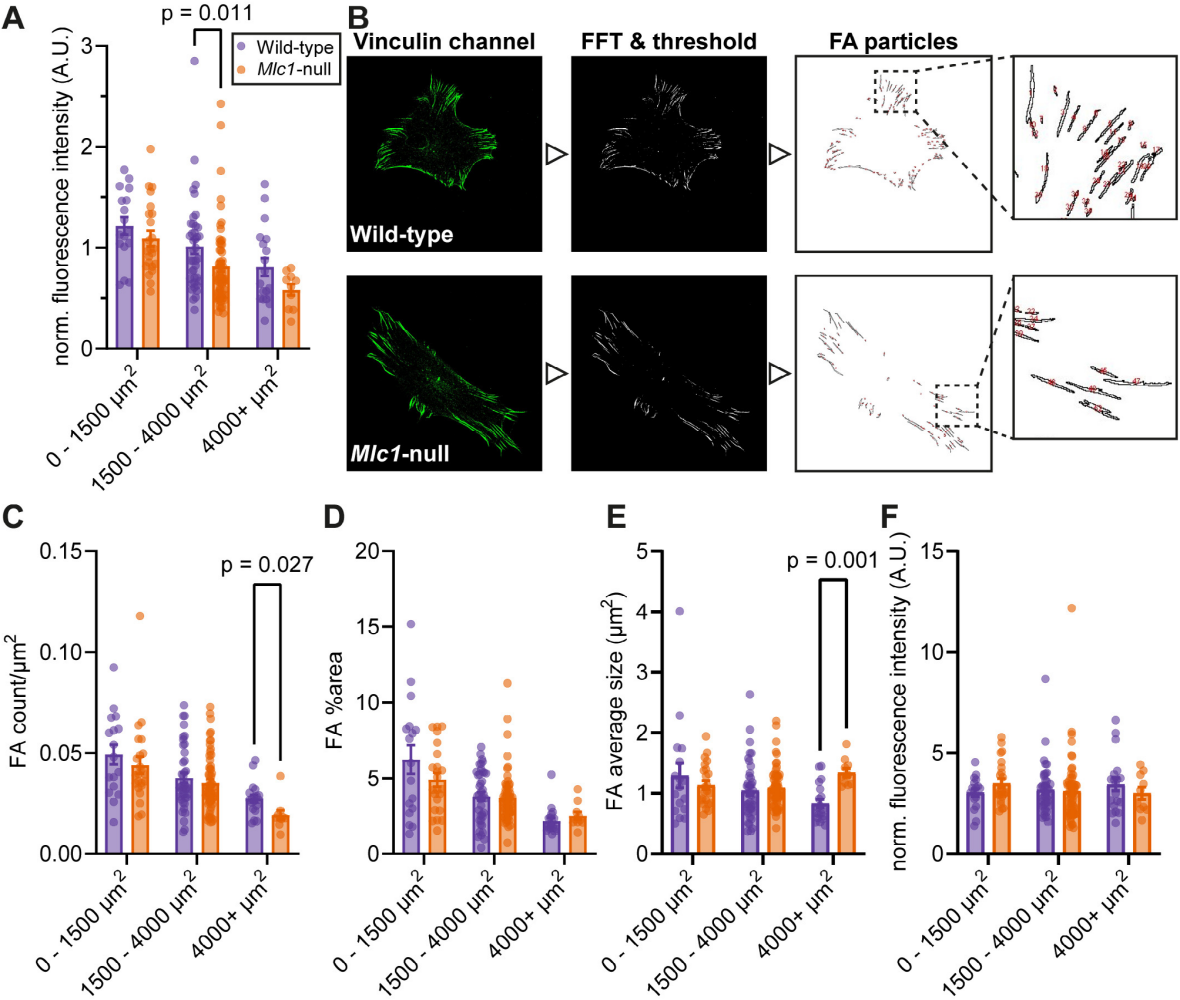
Focal adhesions, represented by vinculin, are altered in *Mlc1*‐null astrocytes. (A) Normalized and background‐corrected fluorescence intensity of vinculin in wild‐type (purple) and *Mlc1‐*null (orange) astrocytes in size groups 0–1500, 1500–4000 and 4000+ μm^2^ (wild‐type 0–1500 μm^2^: 1.22 ± 0.09, *Mlc1*‐null 0–1500 μm^2^: 1.09 ± 0.08, adj. *p* = 0.317, wild‐type 1500–4000 μm^2^: 1.01 ± 0.07, *Mlc1*‐null 1500–4000 μm^2^: 0.81 ± 0.05, adj. *p* = 0.011, wild‐type 4000+ μm^2^: 0.81 ± 0.09, *Mlc1*‐null 4000+ μm^2^: 0.58 ± 0.06, adj. *p* = 0.317). (B) Examples of particle analysis in a wild‐type (first row) and *Mlc1*‐null (second row) astrocyte. Starting with the original MAX projection of the vinculin channel (first image), then the same image after background correction (Fast Fourier Transformation Bandpass Filter) and thresholding (second image). The obtained ROIs of all the focal adhesions (FAs) in the cell (third image) were used to extract different FA parameters. Enlarged ROIs of FAs shown on the right. (C–F) Parameters of FAs in wild‐type (purple) and *Mlc1*‐null (orange) astrocytes in size groups 0–1500, 1500–4000 and 4000+ μm^2^. (C) The number of FAs in the cell per μm^2^ (wild‐type 0–1500 μm^2^: 0.049 ± 0.005, *Mlc1*‐null 0–1500 μm^2^: 0.044 ± 0.004, adj. *p* = 0.585, wild‐type 1500–4000 μm^2^: 0.038 ± 0.002, *Mlc1*‐null 1500–4000 μm^2^: 0.035 ± 0.002, adj. *p* = 0.585, wild‐type 4000+ μm^2^: 0.027 ± 0.002, *Mlc1*‐null 4000+ μm^2^: 0.019 ± 0.002, adj. *p* = 0.027). (D) The percentage of the cell area that contains FAs (wild‐type 0–1500 μm^2^: 6.23% ± 0.95%, *Mlc1*‐null 0–1500 μm^2^: 4.90% ± 0.45%, adj. *p* = 0.695, wild‐type 1500–4000 μm^2^: 3.78% ± 0.28%, *Mlc1*‐null 1500–4000 μm^2^: 3.71% ± 0.22%, adj. *p* = 0.695, wild‐type 4000+ μm^2^: 2.17% ± 0.2%, *Mlc1*‐null 4000+ μm^2^: 2.51% ± 0.26%, adj. *p* = 0.417). (E) The average size of FAs per cell (wild‐type 0–1500 μm^2^: 1.29 ± 0.21 μm^2^, *Mlc1*‐null 0–1500 μm^2^: 1.14 ± 0.07 μm^2^, adj. *p* = 0.914, wild‐type 1500–4000 μm^2^: 1.05 ± 0.08 μm^2^, *Mlc1*‐null 1500–4000 μm^2^: 1.10 ± 0.04 μm^2^, adj. *p* = 0.479, wild‐type 4000+ μm^2^: 0.83 ± 0.07 μm^2^, *Mlc1*‐null 4000+ μm^2^: 1.34 ± 0.07 μm^2^, adj. *p* = 0.001). (F) Normalized and background‐corrected fluorescence intensity of FAs. Individual FA values were averaged per cell (wild‐type 0–1500 μm^2^: 3.04 ± 0.2, *Mlc1*‐null 0–1500 μm^2^: 3.5 ± 0.22, adj. *p* = 0.832, wild‐type 1500–4000 μm^2^: 3.17 ± 0.2, *Mlc1*‐null 1500–4000 μm^2^: 3.1 ± 0.21, adj. *p* = 0.832, wild‐type 4000+ μm^2^: 3.45 ± 0.32, *Mlc1*‐null 4000+ μm^2^: 3 ± 0.3, adj. *p* = 0.832). Each group contains cells from three different mice per genotype (wild‐type 0–1500 μm^2^: *n* = 17/*N* = 3, *Mlc1*‐null 0–1500 μm^2^: *n* = 23/*N* = 3, wild‐type 1500–4000 μm^2^: *n* = 40/*N* = 3, *Mlc1*‐null 1500–4000 μm^2^: *n* = 60/*N* = 3, wild‐type 4000+ μm^2^: *n* = 19/*N* = 3, *Mlc1*‐null 4000+ μm^2^: *n* = 10/*N* = 3). All data is presented as mean ± SEM, with dots indicating individual values of cells in bar graphs.

Patch‐clamp studies on *Mlc1*‐null astrocytes suggested that the cell membrane of these cells was loosely connected to the cytoskeleton. The membrane was pulled further into the patch‐clamp pipette when trying to apply suction, compared to wild‐type astrocytes (Bisseling and Min, personal communication). Therefore, we studied whether the connection of the cytoskeleton to the membrane was altered in *Mlc1*‐null astrocytes. FA complexes connect the actin cytoskeleton to the cell membrane and the ECM through integrin receptors, and can be visualized by immunofluorescence staining for the FA marker vinculin (Burridge et al. 1988). Co‐staining of F‐actin (magenta) and vinculin (green) revealed their partial colocalization in white (Figure 6). In the intermediate‐sized cell group (1500–4000 μm^2^), fluorescence intensity of vinculin was lower in *Mlc1*‐null astrocytes when compared to wild‐type astrocytes (Figure 7A). We quantified the number, morphology and fluorescence intensity of individual FAs by using a particle analysis algorithm (Figure 7C–F; see Figure 7B for examples of analysis) (Güler et al. 2021). Compared to wild‐type astrocytes, the number of FAs per μm^2^ was 30% lower in the largest *Mlc1*‐null astrocytes (4000+ μm^2^; Figure 7C). In this size category we did not observe a change in the percentage of the cell covered by FAs (Figure 7D), which can be explained by a combination of a decrease in the number of FAs, and an increase in FA size (Figure 7E). There was no difference in the fluorescence intensity of FAs between wild‐type and *Mlc1*‐null astrocytes (Figure 7F).

**FIGURE 8:**
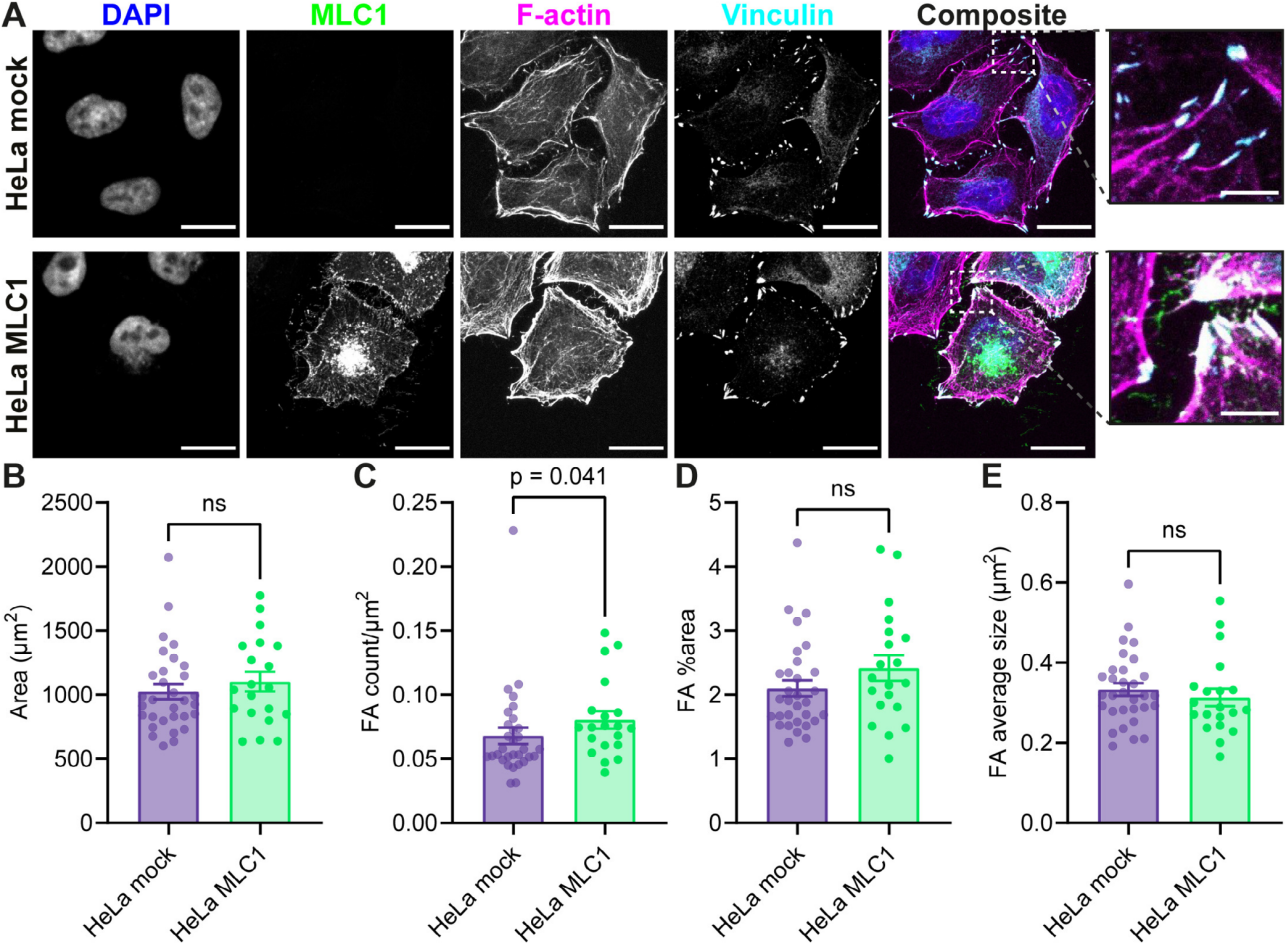
The number of focal adhesions is increased in HeLa‐S3 cells overexpressing MLC1. (A) Representative confocal images of mock‐ and MLC1‐transfected (green) HeLa‐S3 cells, stained for the nucleus with DAPI (blue), F‐actin (magenta) and vinculin (cyan). Focal adhesions (FAs) are shown in the enlarged images. Scale bars: 20 and 5 μm for the enlarged images. (B) Cell area of mock‐transfected (purple) and MLC1‐transfected (green) HeLa‐S3 cells (HeLa mock: 1024 ± 57.97 μm^2^, Hela MLC1: 1102 ± 76.48 μm^2^, *p* = 0.417). (C) The number of FAs in the cell per μm^2^ (HeLa mock: 0.06787 ± 0.006397, HeLa MLC1: 0.08039 ± 0.006796, *p* = 0.041). (D) The percentage of the cell area containing FAs (HeLa mock: 2.1% ± 0.1257%, Hela MLC1: 2.418% ± 0.1974%, *p* = 0.1754). (E) The average size of FAs per cell (HeLa mock: 0.3331 ± 0.01599 μm^2^, HeLa MLC1: 0.3133 ± 0.022 μm^2^, *p* = 0.4608). All data is presented as mean ± SEM, with dots indicating individual values of cells in bar graphs (HeLa mock: *n* = 31 cells, HeLa MLC1: *n* = 20 cells).

Since *Mlc1*‐null astrocytes showed reduced FA numbers, we next asked whether increasing MLC1 levels would have the opposite effect. To this end, we transfected HeLa cells with MLC1 (green) and stained for F‐actin (magenta) and vinculin (cyan) (Figure 8A). Cell size was similar between mock‐ and MLC1‐transfected cells (Figure 8B). MLC1 expression increased the number of FAs per μm^2^ by nearly 20% (Figure 8C), while the fraction of cell area covered by FAs and the average FA size remained unchanged (Figure 8D,E). Together with the reduction of FA numbers in *Mlc1*‐null astrocytes, these results provide complementary evidence that MLC1 directly promotes FA formation.

## 4 Discussion

Dysfunctional astrocytes cause disturbed ion and water homeostasis in MLC, but the exact mechanism of this disturbance is not fully understood. Here, we use different approaches to demonstrate that the mechanical properties of primary astrocytes are altered upon loss of MLC1. Using a specialized indentation method, we show that *Mlc1*‐null astrocytes are softer than wild‐type astrocytes. Proteomic and western blot analysis of *Mlc1*‐null astrocytes reveals alterations in several cytoskeleton‐related pathways. Confocal imaging of cytoskeletal organization in *Mlc1‐*null astrocytes indicates no changes in actin cytoskeleton structure or microtubule acetylation. Instead, we observe a reduction in FA numbers in *Mlc1*‐null astrocytes and the opposite effect in HeLa cells overexpressing MLC1, together supporting the idea that MLC1 regulates FA formation.

We use indentation experiments to show that *Mlc1*‐null astrocytes are soft. Cytochalasin D (Brenner and Korn 1979), an actin polymerization inhibitor that causes a near‐complete collapse of the actin cytoskeleton, makes wild‐type astrocytes softer but does not induce further softening in *Mlc1*‐null astrocytes. Since actin is the main contributor to cell stiffness in astrocytes (Curry et al. 2017), we investigate whether reduced cell stiffness in *Mlc1*‐null astrocytes could be due to disruption of the actin cytoskeleton. However, imaging shows that the actin cytoskeleton is morphologically intact in *Mlc1*‐null astrocytes.

Proteomic analysis and western blots reveal that *Mlc1*‐null astrocytes show differential expression of proteins implicated in cytoskeleton‐related pathways. We observe differential expression of several actin isoforms, but immunofluorescent staining does not reveal obvious alterations in the intensity or gross distribution of the actin cytoskeleton. As phalloidin stains all actin isoforms, there might be a shift in actin isoforms that is not picked up in our immunofluorescence experiments. In addition to proteins related to the actin cytoskeleton, several microtubule‐related proteins are altered. Microtubules are involved in intracellular transport, cell division and structural support (Weigel et al. 2021), and play a role in mechanotransduction (Seetharaman et al. 2022). Acetylated microtubules represent the stable microtubules pool. Overall, we do not find differences in microtubule acetylation between *Mlc1*‐null and wild‐type astrocytes.

The question remains what causes the softening of *Mlc1*‐null astrocytes. Whilst performing patch‐clamp experiments, we noticed that the membranes of *Mlc1*‐null astrocytes were pulled further into the pipette when generating a gigaseal (Bisseling and Min, personal communication). This observation could not be quantified but suggests a compromised attachment of the membrane to the cytoskeleton. This prompted us to investigate cytoskeleton‐membrane‐ECM interactions. We focused on FAs, large protein complexes that connect the actin cytoskeleton, membrane and ECM (Legerstee and Houtsmuller 2021). In our immunofluorescence experiments, staining for the FA protein vinculin reveals changes in FA number and size in *Mlc1*‐null astrocytes, even though the vinculin protein level is unaltered in proteomics and western blotting. As a large pool of inactive vinculin in closed conformation resides in the cytosol, the total vinculin protein levels do not necessarily reflect changes related to FAs. Previous studies have established that loss of vinculin reduces cell stiffness and decreases adhesion to the ECM (Goldmann et al. 1998; Mierke et al. 2008, 2010; Klemm et al. 2009). Changes in FAs in *Mlc1*‐null astrocytes might therefore lead to a reduced tensile strength on the actin cytoskeleton, resulting in softer *Mlc1*‐null astrocytes. To complement this finding, we tested the opposite condition by transfecting HeLa cells with MLC1, which results in an increased number of FAs. Together, these observations support the idea that MLC1 influences FAs and thereby astrocyte mechanical properties.

FAs are highly dynamic structures, and their turnover is essential for cell motility and migration (Yamaguchi and Knaut 2022). Tension‐induced activation of vinculin stabilizes and matures FAs, which enforces the cytoskeleton‐membrane‐ECM interaction and enables cells to endure mechanical tension (Galbraith et al. 2002; Grashoff et al. 2010; Carisey et al. 2013; Dumbauld et al. 2013). As FA dynamics determine the strength of this interaction, an exciting next step would be to compare FA dynamics in wild‐type and *Mlc1*‐null astrocytes using live‐cell imaging. Interestingly, THY‐1 is upregulated in *Mlc1*‐null astrocytes. This is a glycoprotein that binds integrin receptors in astrocytes and promotes FA formation and cell spreading (Leyton et al. 2001). An explanation might be that FAs are formed but not maintained in *Mlc1*‐null astrocytes, which is supported by a decrease in expression of essential FA component integrin β5 (Chastney et al. 2025). This would lead to high turnover and less stable FAs and an increase in THY‐1 levels. Such dynamic changes could be determined with live‐cell imaging.

Cultured primary astrocytes are a useful tool to understand the cell biology of MLC1. However, they do not replicate in vivo morphology. It is therefore crucial to explore whether cytoskeletal and mechanical properties of intact astrocytes in the brain are altered in MLC. Of particular interest is studying the mechanical properties of perivascular astrocyte endfeet, where MLC1 is highly expressed (Boor et al. 2005). These structures are anchored to the vasculature through interaction of membrane proteins with the ECM. Our data suggest a compromised cytoskeleton‐membrane‐ECM interaction upon loss of MLC1. In line with this, an earlier study established that absence of MLC1 leads to disturbed adhesion of endfeet to the vasculature (Gilbert et al. 2021). ECM proteins form the perivascular basal lamina to which the endfoot adheres. Notably, a loss of ECM proteins can lead to brain abnormalities that resemble MLC (Min and van der Knaap 2018). Pathogenic variants in one such protein, laminin α2, lead to Merosin‐deficient Congenital Muscular Dystrophy (CMD) (Helbling‐Leclerc et al. 1995), and MRI abnormalities of the brain white matter in these patients display striking similarities with MLC (Philpot et al. 1995; van der Knaap et al. 1997). The DAGC, which binds laminin in the ECM, is colocalized with MLC1 in astrocyte endfeet (Boor et al. 2007; Ambrosini et al. 2008). A specialized structure known as the costamere, composed of the DAGC and FAs, is present in striated muscle cells (where MLC1 is not expressed), where it regulates cytoskeleton‐membrane‐ECM interactions (Jaka et al. 2015). We hypothesize that MLC1 is part of an analogous structure in astrocyte endfeet that regulates these interactions. Future studies to validate such a structure in perivascular endfeet in MLC mouse tissue, with super‐resolution imaging, could provide valuable insights into MLC pathology, as well as astrocyte physiology.

An open question is how disrupted mechanical properties of astrocyte endfeet lead to brain edema. Many studies on MLC have provided evidence for (mechanosensitive) ion channel dysfunction and disturbed volume regulation in MLC (Ridder et al. 2011; Jeworutzki et al. 2012; Lanciotti et al. 2012; Passchier et al. 2023). The function of ion and water channels involved in volume regulation is very much dependent on cytoskeleton‐membrane‐ECM interactions. For example, VRAC is modulated by membrane stretch and the cytoskeleton (Byfield et al. 2006), and chloride currents similar to VRAC activation can be evoked by stretching of integrin receptors (Browe and Baumgarten 2006). TRPV4 is another important mechanosensitive ion channel implicated in astrocyte volume regulation (Benfenati et al. 2007), and its function strongly depends on its association with the cytoskeleton (Becker et al. 2009). Finally, AQP4 localization is modulated by the actin cytoskeleton (Nicchia et al. 2008). It is likely that alterations in astrocyte mechanical properties contribute to dysregulation of ion channels and thereby lead to defective volume regulation in MLC. In addition, astrocyte endfeet form a crucial component of the so‐called glymphatic system (Jessen et al. 2015). Recent computational studies have shown that endfeet might act as ‘valves’ around the vasculature to regulate perivascular fluid flow (Bork et al. 2023; Gan et al. 2023, 2024). Thus, a disruption of the structural integrity of endfeet in MLC might directly alter glymphatic fluid flow by interfering with such a valve function.

Interplay between MLC1 or GlialCAM and the cytoskeleton is also observed outside of astrocyte endfeet. Astrocyte specializations that point towards the synapse, so‐called perisynaptic processes, also contain MLC1. In *Mlc1*‐null mice, these processes are retracted from synapses due to a shortened tip length (Kater et al. 2023). This phenotype resembles what is seen in astrocytes lacking ezrin (Badia‐Soteras et al. 2023), an actin‐membrane linker that regulates FA dynamics (Hoskin et al. 2015). MLC1 and GlialCAM have been shown to regulate cell motility and proliferation in various cell lines, and dysregulation of these proteins, as well as their newly identified interaction partner GPRC5B (Alonso‐Gardón et al. 2021; Passchier et al. 2023), has been implicated in cancer (Lanciotti et al. 2016; Hwang et al. 2019; Lattier et al. 2020; Kanamori et al. 2023; Wu et al. 2024). MLC1 can regulate actin dynamics by interacting with the Arp2/3 complex (Hwang et al. 2019), which drives lamellipodia protrusion through actin networks advancing at the membrane (Swaminathan et al. 2016; Gautreau et al. 2022). GlialCAM is essential for cell‐ECM adhesion (Moh et al. 2005), and regulates FA signaling pathways in glioma (De et al. 2022). These studies show that MLC1 and its interaction partners regulate cytoskeleton‐related processes in several physiological and pathological conditions.

In conclusion, our study provides a new perspective on astrocyte dysfunction in MLC. We present evidence for a role of MLC1 in regulating astrocyte mechanobiology. Our results provide a basis for future investigations into how MLC1 is involved in tuning the mechanical properties of intact astrocytes, how this relates to astrocyte volume regulation and brain fluid dynamics, and how disruption of these processes leads to chronic brain edema in MLC.

## Author Contributions

Study design: Q.B., E.M.J.P. and R.M. Indentation experiments and analysis: Q.B., E.M.J.P. and N.A. Proteomic and western blot experiments and analysis: Q.B., F.M.K., S.C., M.S.B. and E.A. Immunofluorescence experiments and analysis: Q.B., F.M.K. and A.F. Provision of resources and funding: H.D.M., M.S.K. and R.M. Figures: Q.B. and S.C. Writing manuscript: Q.B. and R.M., with feedback from all authors.

## Funding

This study was supported by a ZonMw Vidi grant (91718392 to R.M.) and the Dutch Rare Disease Foundation (Zeldzame Ziekten Fonds).

## Ethics Statement

Experimental procedures involving mice were in strict compliance with animal welfare policies of the Dutch government and were approved by the Institutional Animal Care and Use Committee of the Amsterdam University Medical Center, location AMC, Amsterdam, or of the Vrije Universiteit Amsterdam, depending on the location of the experiments.

## Conflicts of Interest

The authors declare no conflicts of interest.

## Supporting information


**Figure S1:** Selected proteins for western blot analysis based on proteomic findings in Figure 2. Bar plot of log_2_ fold changes of protein expression in *Mlc1*‐null astrocytes compared to wild‐type astrocytes with upregulated (red), downregulated (blue), and not changed (gray) proteins, based on a fold change > 0.06 or < −0.06 with an adj. *p* < 0.05.
**Figure S2:** Original western blots of Figure 3. Panel letters correspond with the panel letters in Figure 3, and red boxes outline the western blot images used in Figure 3. Molecular weight (MW) markers in kDa are indicated on the left. GAPDH was used as a loading control.

**Data S1:** Supporting Information.

**Data S2:** Supporting Information.

## Data Availability

Data supporting the findings from this study is available from the authors upon reasonable request.
