# Supplementary material for: Altered Mechanical Properties of Astrocytes Lacking MLC1: Implications for the Leukodystrophy MLC
Source: Glia. 2025 Dec 2;74(2):e70104. doi: 10.1002/glia.70104 (PMC12672979; doi:10.1002/glia.70104)
Supplement: Supplementary file 1 — Figure S1: Selected proteins for western blot analysis based on proteomic findings in Figure 2. Bar plot of log2 fold changes of protein expression in Mlc1‐null astrocytes compared to wild‐type astrocytes with upregulated (red), downregulated (blue), and not changed (gray) proteins, based on a fold change > 0.06 or < −0.06 with an adj. p < 0.05. Figure S2: Original western blots of Figure 3. Panel letters correspond with the panel letters in Figure 3, and red boxes outline the western blot images used in Figure 3. Molecular weight (MW) markers in kDa are indicated on the left. GAPDH was used as a loading control. [file GLIA-74-0-s003.pdf]

# SUPPLEMENTARY

## Supplementary figures

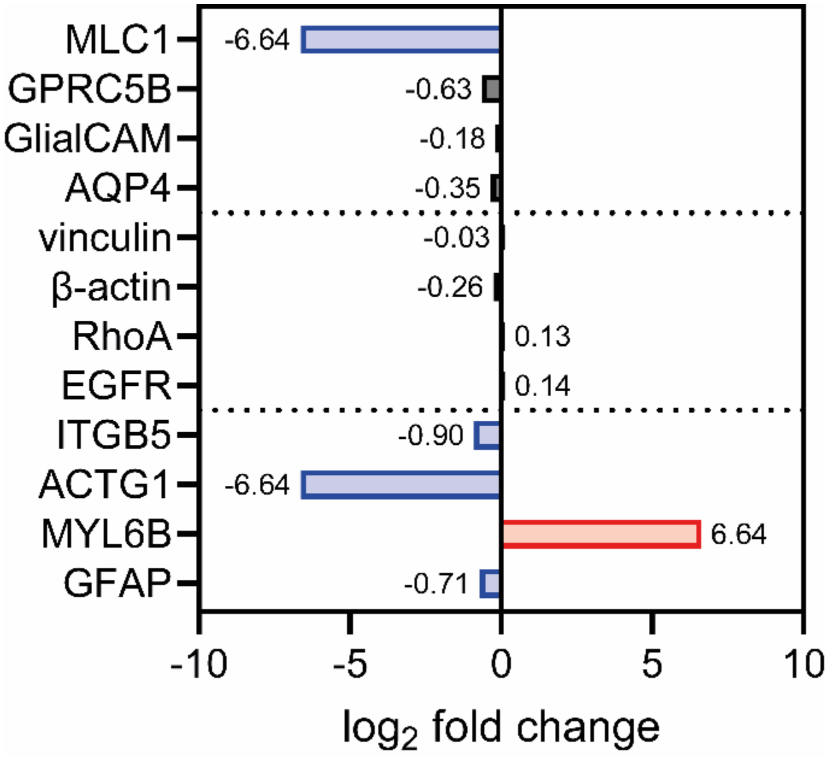


**Figure S1. Selected proteins for western blot analysis based on proteomic findings in Figure 2**. Bar plot of $log_2$ fold changes of protein expression in *Mlc1*-null astrocytes compared to wild-type astrocytes with upregulated (red), downregulated (blue), and not changed (grey) proteins, based on a fold change > 0.06 or < -0.06 with an adj. *P*<0.05.

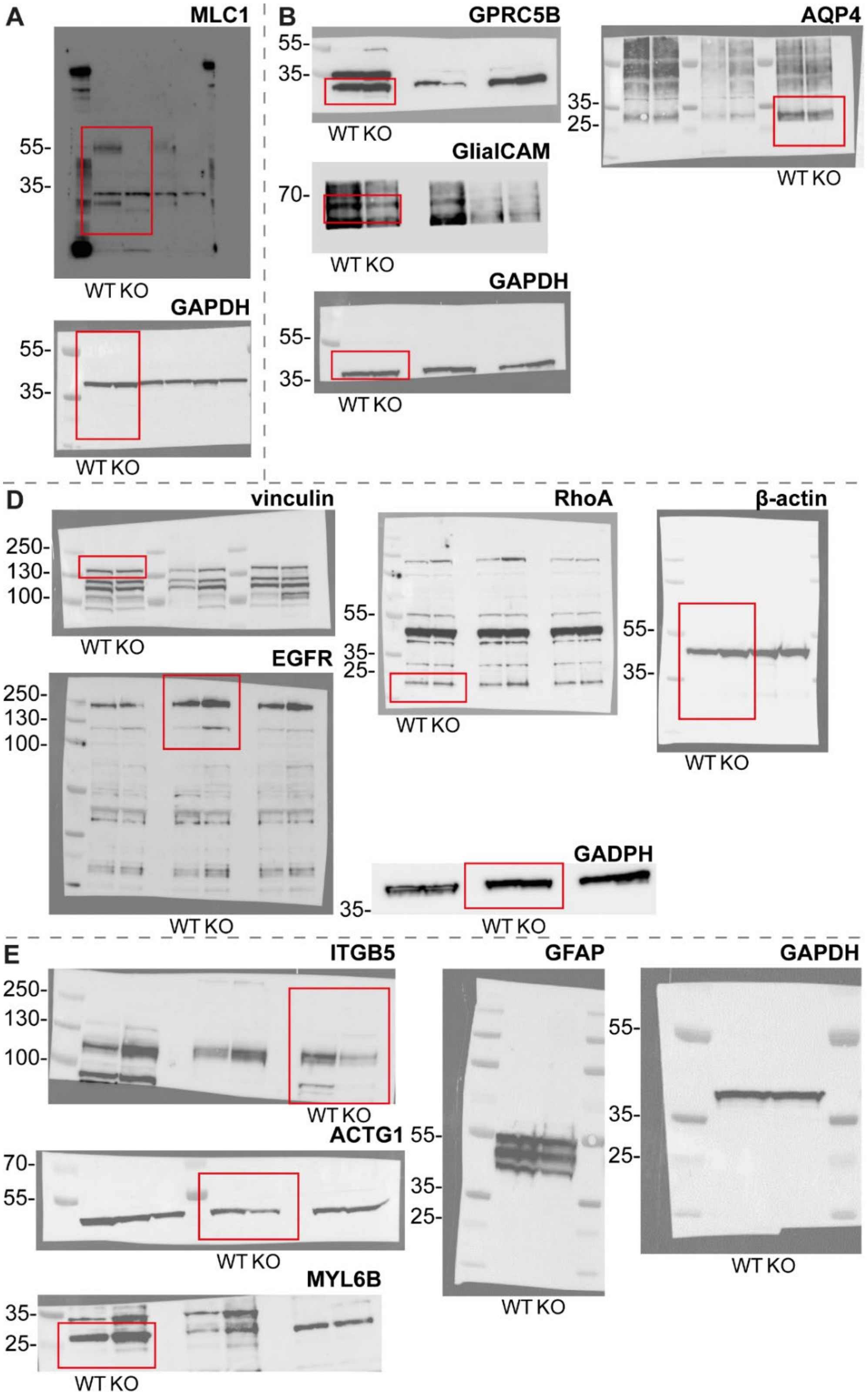


**Figure S2. Original western blots of Figure 3.** Panel letters correspond with the panel letters in Figure 3, and red boxes outline the western blot images used in Figure 3. Molecular weight (MW) markers in kDa are indicated on the left. GAPDH was used as a loading control.
